# Genetic determinants of cannabis use: a systematic review protocol

**DOI:** 10.1186/s13643-020-01442-2

**Published:** 2020-08-20

**Authors:** Alannah Hillmer, Caroul Chawar, Stephanie Sanger, Alessia D’Elia, Mehreen Butt, Raveena Kapoor, Flavio Kapczinski, Guillaume Pare, Lehana Thabane, Zainab Samaan

**Affiliations:** 1 Neuroscience Graduate Program, Department of Psychiatry and Behavioural Neurosciences, McMaster University, 100 West 5th St., Hamilton, ON L8N 3 K7 Canada; 2 Health Science Library, McMaster University, 1280 Main St. W, Hamilton, ON L8S 4 L8 Canada; 3 Integrated Science Program, McMaster University, 1280 Main St. W, Hamilton, ON L8S 4 L8 Canada; 4 Health Sciences Program, McMaster University, 1280 Main St. W, Hamilton, ON L8S 4 L8 Canada; 5 Department of Psychiatry and Behavioural Neurosciences, McMaster University, 100 West 5th St., Hamilton, ON L8N 3 K7 Canada; 6 Population Health Research Institute, McMaster University, 1280 Main St. W, Hamilton, ON L8S 4 L8 Canada; 7 Department of Health Research Method, Evidence & Impact, 1280 Main St. W, Hamilton, ON L8S 4 L8 Canada

**Keywords:** Systematic review, Cannabis, Genetics, Genome-wide

## Abstract

**Background:**

With the legalization of cannabis in Canada, there is an increase trend in use. Cannabis has been known to have several health implications, one of which is the development of cannabis use disorder (CUD). CUD is more common in males than females, as well as in certain ethnic groups such as Native Americans. Additionally, both environmental and genetic risk factors have been found for cannabis use. The objective of this systematic review will be to summarize the genetic variants associated with cannabis use which have reached borderline genome-wide significance.

**Methods:**

This systematic review will incorporate articles that have performed a genome-wide association study (GWAS) investigating cannabis use. MEDLINE, Web of Science, EMBASE, GWAS Catalog, GWAS Central, and NIH Database of Genotype and Phenotype will be searched using a comprehensive search strategy. The quality of genetic association studies (Q-Genie) tool will be utilized to assess the quality of the included studies. All screening and data extraction will occur independently by two authors. If feasible, a random-effects meta-analysis will be conducted on pooled odds ratios of single nucleotide polymorphisms reaching borderline genome-wide significance.

**Discussion:**

This systematic review will synthesize available GWAS on cannabis use. Results from this review will inform and direct further investigation of genetic variants associated with cannabis use.

**Systematic review registration:**

PROSPERO CRD42020176016

## Background

On October 17, 2018, the Cannabis Act came into effect in Canada allowing for the legal growth of cannabis plants as well as the recreational possession and consumption of cannabis for those who are 18 years or older [1]. In response to the Cannabis Act, Statistics Canada has introduced a National Cannabis Survey which has been conducted every 3 months since February 2018. The NCS showed that nearly 17% of Canadians aged 15 years and older reported using cannabis within a 3-month period between mid-August and mid-September of 2019, a rate that was consistent with the rate of the year prior, when cannabis was an illicit substance. However, in the fourth quarter of 2019, cannabis use was increased when compared to the fourth quarter of 2018. Additionally, regardless of the year of study, cannabis consumption rates continue to be higher among males than females [2].

Cannabis use disorder (CUD) is defined as a problematic pattern of cannabis use leading to clinically significant impairment or distress. In 2013, the Diagnostic and Statistical Manual reported that CUD is prevalent in 3.4% of youth aged 12 to 17 years old and 1.5% of adults age 18 years or older. Trends of CUD also differ among sex and ethnicities. Rates of CUD are higher in males compared to females and rates of CUD are higher in Native American and Alaska Natives compared to other ethnic groups [3]. Results from a meta-analysis on twin studies estimated the heritability for cannabis use initiation to be 40–48% and 51–59% for problematic cannabis use, suggesting a genetic component to cannabis use and CUD [4]. A genome-wide association study (GWAS) combined five cohorts identifying several genes and single nucleotide polymorphisms (SNPs) associated with cannabis use and dependence [5]. A cluster of correlated SNPs in a novel region of chromosome 10 were identified at genome-wide significant levels in participants of European descent [5]. However, of three meta-analyses conducted on cannabis use in the literature, only one study identified a significate association [6–8]. One region on chromosome 16 was significantly associated with age of first cannabis use, with the strongest association for the intronic variant rs1574587 [7].

Interestingly, one study investigated the genetic and environmental risk factors for cannabis availability reported variation in cannabis initiation and symptoms of cannabis use disorder. Cannabis availability and initiation had a correlation of 0.48 and cannabis availability and symptoms of cannabis use disorder had a correlation of 0.23. Additionally, much of the variation associated with problematic use can be explained by shared environmental risk in cannabis availability leading to initiation and the genetic non-shared environmental risks for cannabis initiation [9]. These findings are of specific interest to Canada and other countries with legalization of cannabis is already in effect or being considered, as cannabis is increasingly more available since the legalization.

With cannabis availability increasing, and known heritability of CUD, it is important to understand the genetic risk factors associated with cannabis use. While meta-analyses of GWASs provide regions of interest, no known systematic review exists that summarizes identified genes and/or SNPs that have reached genome-wide significance for cannabis use. It is important to provide a summary of the literature which includes recent GWASs in the context of cannabis legalization. Further, understanding the genetic basis of cannabis use will assist health care workers in making science-informed decisions regarding the recommendation of recreational use and prescription of cannabis.

### Objectives

The main goal of this systematic review is to identify genetic variants from genome-wide association studies (GWASs) associated with cannabis use. Though genetic variants most commonly reported by GWASs are SNPs, this review will be inclusive of any other genetic markers reported in GWASs. We will summarize the results of GWASs which meet our inclusion criteria, and if possible, we will meta-analyze genetic variants that are reported in more than one primary study.

Primary objectives of this systematic review include the following:

1. Identify genetic variants associated with current cannabis use. Current cannabis use is defined by either self-report or positive urine drug screens within 1 month of the study being conducted.
2. Identify genetic variants associated with lifetime cannabis use. Lifetime cannabis use is defined by any self-reported or positive urine drug screens of cannabis use within one’s lifetime.
3. Identify genetic variants associated with CUD. CUD is defined by any diagnostic and classification systems used to diagnosis CUD or questionnaires validated to assess CUD.

Secondary objectives of this systematic review include the following:

1. Identify genetic variants associated with the adverse outcomes of cannabis use including psychiatric (cognitive impairment, psychotic symptoms, depression, anxiety, suicidal behavior) and non-psychiatric (chronic bronchitis, lung infections, chronic cough, increased risk of motor vehicle accidents) [10–12].
2. When feasible, perform subgroup summaries by sex or ethnic differences.

## Methods and analysis

This protocol is reported in accordance with the Preferred Reporting Items for Systematic Reviews and Meta-Analyses Protocols (PRISMA-P) statement [13] (see PRISMA-P checklist in Additional file 1). This protocol was registered within the International Prospective Register of Systematic Reviews (PROSPERO) (registration number: CRD42020176016).

### Eligibility criteria

GWAS studies presenting original data on associations between cannabis use and genetic polymorphisms using any study design (i.e., case-control, cohort, etc.) will be included in this systematic review. All other types of studies will be excluded. Studies in any setting will be included and no restriction will be placed on age, sex, ethnic background, or language. Additionally, articles that do not present sufficient data to calculate the odds ratio (OR) with a 95% confidence interval will be excluded from quantitative analyses if data cannot be obtained after contacting the studies’ authors and the calculations cannot be made with the available published information. However, we will include these studies in the qualitative description of the review findings.

We will include studies investigating cannabis use disorder as defined by the Diagnostic and Statistical Manual-5 (DSM-5) or other diagnostic and classification systems such as the International Statistical Classification of Diseases and Related Health Problems-10 (ICD-10) or specific diagnostic scales designed to screen and diagnose dependence or use disorder of cannabis as well as any studies measuring any use of cannabis. We define cannabis use based on the included studies’ definitions and accept the following definition: current cannabis use is defined as either self-report or positive urine drug screens within 1 month of the study being conducted and lifetime cannabis use is defined as any self-reported or positive urine drug screens of cannabis use within one’s lifetime [14]. Clinical diagnoses and questionnaires validated to assess CUD will also be accepted. All studies not investigating current cannabis use, lifetime cannabis use, or CUD will be excluded. In the case of polymorphisms reported in duplicate publications from the same study population, the article that is the most recent will be included.

### Information sources

A Health Science Librarian was consulted to develop a comprehensive search strategy. No language restriction will be placed on the search strategy, though studies will be limited to human studies. MEDLINE, Web of Science, EMBASE, GWAS Catalog, GWAS Central, and NIH Database of Genotype and Phenotype will be searched using the agreed-upon strategy, modified for each database. The search strategy will include all terms relevant to cannabis and genome-wide association studies. Databases will be searched from inception onwards. Sources of gray literature including dissertations and theses, clinical guidelines, and reports from regulatory agencies will be searched. Reference lists of relevant systematic reviews and all included studies will be checked to identify additional articles.

### Search strategy

Draft search strategies for multiple electronic databases are provided in Additional file 2.

### Study records

#### Data management

All of the references will be managed and organized through Zotero [15]. Covidence will be used for the management of this systematic review at the title and abstract, full text, and data extraction stages [16]. Prior to the formal screening process, a calibration will take place to pilot and refine the screening process. Training will be given to all team members on using Covidence prior to starting the review.

#### Selection process

Two independent reviewers will screen titles and abstracts for inclusion criteria. Full-text review will also be completed independently by two reviewers. Disagreements between reviewers will be resolved by consensus or including a third reviewer. We will record the reason for excluding studies at the full-text review stage.

#### Data collection process

Data extraction will take place independently and in duplicate for each eligible study. Standardized full-text data extraction forms will be constructed. The data extraction form will be pilot tested by two independent reviewers to determine the feasibility of this review and ensure all details are captured. In the event of missing data, we will contact study authors to obtain missing information where possible. All contact with the authors will be documented.

#### Data items

We will extract the following information: author, year of study, country, cohort population used, number of participants (separated by those included in the cannabis use group and non-cannabis use group), control population, the ethnicity of participants, mean age, sex ratio, the measure of cannabis use disorder or cannabis use or definition of cannabis use, inclusion and exclusion criteria, how cannabis use was reported (i.e., self-report, drug urine screens), frequency of cannabis use, and finally any genetic variants which reached the significance threshold set of *p* ≤ 10^−7^. Genome-wide significance is generally considered any SNP with a *p* value less than 5 × 10^−8^; however, SNPs reaching borderline significance, *p* < 10^−7^, will also be extracted as borderline significance has been found to be generally replicable [17].

### Outcomes and prioritization

The main aim of the systematic review will be to assess variants reaching the given threshold associated with cannabis use outcomes from the primary studies included in this review.

The primary outcomes are as follows:

1. Current cannabis use is defined as either self-reported cannabis use or positive cannabis urine drug screens within 1 month of the study being conducted.
2. Lifetime cannabis use is defined as self-reported ever used cannabis during the individual’s lifetime.
3. CUD is defined by a diagnosis from the DSM-5 or other diagnostic and classification system such as the ICD-10 or specific diagnostic scales designed to screen and diagnose dependence or use disorder of cannabis.

For each of the outcomes above, we will collect information on each outcome as reported in the primary studies meeting the eligibility criteria, including dichotomous use of cannabis, percent positive urine screens, questionnaires, and diagnostic classification.

The secondary outcomes are as follows:

1. Adverse outcomes of cannabis use including psychiatric and non-psychiatric outcomes. We will collect data as reported in the primary studies included such as comorbid diagnosis and additional medication condition.
2. We will collect information from the included primary studies on sex and ethnic groups within the study. We will provide a qualitative summary and, if feasible, conduct a subgroup meta-analysis of genetic variants within specific ethnic groups.

### Risk of bias in individual studies

Quality assessment will be completed in duplicate for each study included. The quality of genetic association studies (Q-Genie) tool [version 1.1] will be used. Disagreements of quality assessments will be resolved through discussion [18]. If a consensus is not reached through discussion, a third author will be consulted to resolve the disagreement.

### Data synthesis

Studies included in this systematic review will undergo qualitative synthesis. Summary tables will be used which will include the sample size, size of cannabis group and non-cannabis group, sex distribution, mean age, study design, ethnic population, and outcome (current cannabis use, lifetime cannabis use, or CUD). A separate table will be used to display any variants reaching borderline genome-wide significance, the corresponding study it was reported in, the corresponding chromosome and position, minor allele, gene/locus, population size, outcome associated, measure, measure of association value, measure of variability, ethnicity, and *p* value reported.

Heterogeneity between the studies will be assessed through the *I*^2^ statistic with a 95% confidence interval. We will also report summary tables including the study design, population, and cannabis use measure/definition to describe heterogeneity qualitatively. If appropriate, a random-effects meta-analysis will be conducted on pooled odds ratios for the main outcome previously mentioned. If appropriate, the a random-effects meta-analysis will be conducted on pooled odds ratio for the secondary outcomes previously mentioned as well as a subgroup analyses of the participants’ sex and ethnicities. Subgroup analyses by participant’s sex account for any differences in cannabis use between sexes which has been previously reported in the literature [19–21]. Additionally, due to genetic differences between ethnicities, genetic associations may be more predominant in certain ethnic groups than others, as such a subgroup analysis will be conducted, if feasible [22]. Studies excluded from the quantitative analysis will be listed and an exclusion reason will be given.

If quantitative methods of analysis are not feasible for both the primary or secondary outcomes due to either low heterogeneity found by the *I*^2^ statistic or qualitative synthesis or no two study reports the same genetic variant, only qualitative synthesis results will be reported. We will not conduct a meta-analysis of individual participant data.

### Meta-bias

To help mitigate publication bias conference, abstracts will included, manual searches of references lists will be conducted, and Cochrane Clinical Trail Protocols Registry and ClinicalTrails.gov databases will be searched for relevant clinical trial protocols. Additionally, the GWAS catalog will be manual searched for borderline significant variants associated with current cannabis use, lifetime cannabis use, or CUD to ensure all variants are captured within this review. Authors of conference abstracts will be contacted to determine the stage of the research project and all correspondence will be documented. If the published work was not captured by the search strategy and deemed eligible by two independent reviewers, it will be included. Two independent reviewers will search the references lists of all included studies. Any identified references, deemed eligible by two independent reviewers, will be included.

### Confidence in cumulative estimate

The Grading of Recommendations Assessment, Development and Evaluation (GRADE) will be used to assess the strength of evidence. GRADE scores according to the risk of bias, publication bias, consistency, directness, and precision. A score of high-, moderate, low-, or very low-quality evidence will be assigned and summarized in a table [23].

### Presenting and reporting of results

The full review will follow the Preferred Reporting Items for Systematic Reviews and Meta-Analysis (PRISMA) guidelines with special consideration to the Human Genome Epidemiology Network (HuGENet) guidelines [24]. Although HuGENet reviews typically focus on a single gene, we will present information on each genetic variant-phenotype association reported which will include the study details, population, findings, and source of data.

## Discussion

A lack of consistent evidence exists in the current literature for genetic variants associated with cannabis use. In addition, this is the first known systematic review to synthesize the available evidence on genetic variants associated with cannabis use. The proposed systematic review aims to identify all genetic variants that have reached borderline genome-wide significance associated with cannabis use and CUD. The proposed systematic review will provide an overview of the current literature on the genetics of cannabis, aiding in the genetic understanding of cannabis use. Understanding the genetic contribution to cannabis use and its effects such as cannabis use disorder has the potential to aid medical practitioners in making decisions related to cannabis use for medical reasons and the associated potential risks. Additionally, variants reaching borderline genome-wide significance will be examined in the context of their known or biologically plausible relevance to further our understanding.

Anticipated limitations of this review existed at both the study and review level. Limitations at the study level may include a lack of reporting quality control steps, reporting of variants within linkage disequilibrium, small sample size, and a lack of reporting variants that failed to reach genome-wide significance (*p* < 5 × 10^−8^) but may have reached borderline significance levels (*p* < 10^−7^). At the review level, limitations exist in the expected high heterogeneity, differing outcomes for cannabis use reported in the literature and the exclusion of meta-analysis and candidate gene studies.

On completion of the systematic review, we will publish in a peer-review academic journal to reach both clinical and academic experts in the field. This systematic review will then inform and direct the further investigation of genetic variants associated with cannabis through candidate gene studies.

## Supplementary information

**Additional file 1.** “PRISMA-P 2015 Checklist” and contains PRISMA-P checklist.

**Additional file 2.** Search strategy.

## Data Availability

Data sharing is not applicable to this article as no datasets were generated or analyzed during the current study.

## Abbreviations

CUD: Cannabis use disorder
DSM-5: Diagnostic and Statistical Manual 5th edition
GRADE: Grading of Recommendations Assessment, Development and Evaluation
GWAS: Genome-wide association study
GWASs: Genome-wide association studies
HuGENet: The Human Genome Epidemiology Network
ICD-10: International Statistical Classification of Diseases and Related Health Problems -10
PRISMA: Preferred Reporting Items for Systematic Reviews and Meta-Analyses
PRISMA-P: Preferred Reporting Items for Systematic Reviews and Meta-Analyses Protocols
Q-Genie: The quality of genetic association studies
