# Supplementary material for: Genetic determinants of cannabis use: a systematic review protocol
Source: Syst Rev. 2020 Aug 20;9:190. doi: 10.1186/s13643-020-01442-2 (PMC7441561; doi:10.1186/s13643-020-01442-2)
Supplement: Supplementary file 2 — Additional file 2. Search strategy. [file 13643_2020_1442_MOESM2_ESM.docx]

| Search strategy | |
| --- | --- |
| MEDLINE | 1. Genome-Wide Association Study/ 2. Genotyping Techniques/ 3. Genome, Human/ 4. Genetic Variation/ 5. genetics/ or exp human genetics/ 6. (human* adj2 (genotyp* or genome* or genetic*)).ti,ab,kw,kf. 7. (GWS or GWAS or GWA).mp. 8. genome wide.ti,ab,kw,kf. 9. 1 or 2 or 3 or 4 or 5 or 6 or 7 or 8 10. exp Cannabis/ 11. ((cannabis* or marijuana* or cannabinoids* or marihuana* or hash* or kush* or weed* or pot* or THC* or CBD*) adj2 (overdose* or use* or using or misuse* or abus* or dependence* or addict*)).ti,ab,kw,kf. 12. 10 or 11 13. 9 and 12 14. *Limit 13 to humans* |
| Web of Science | 1. TS=(genome-wide association study or genome-wide association or GWAS or GWA or genome wide) 2. TS=(human NEAR/2 genome) 3. TS=(( cannabis* or marijuana* or cannabinoids* or marihuana* or hash* or kush* or weed* or pot* or THC* or CBD*) NEAR/2 (overdose* or use* or using or misuse* or abus* or dependence* or addict*)) 4. TS=(cannabis* or marijuana* or marihuana*) 5. #1 OR #2 6. #3 OR #4 7. #5 and #6 |
| EMBASE | 1. Genome-Wide Association Study/ 2. Genotyping Techniques/ 3. Genome, Human/ 4. Genetic Variation/ 5. genetics/ or exp human genetics/ 6. (human* adj2 (genotyp* or genome* or genetic*)).ti,ab,kw. 7. (GWS or GWAS or GWA).mp. 8. genome wide.ti,ab,kw. 9. 1 or 2 or 3 or 4 or 5 or 6 or 7 or 8 10. exp Cannabis/ 11. ((cannabis* or marijuana* or cannabinoids* or marihuana* or hash* or kush* or weed* or pot* or THC* or CBD*) adj2 (overdose* or use* or using or misuse* or abus* or dependence* or addict*)).ti,ab,kw. 12. 10 or 11 13. 9 and 12 14. *Limit 13 to human* |
| CINAHL | 1. genome-wide association study or genome-wide association or GWAS or GWA or genome wide or genome 2. cannabis* or marijuana* or cannabinoids* or marihuana* or hash* or kush* or weed* or pot* or THC* or CBD*) 3. overdose* or use* or using or misuse* or abus* or dependence* or addict* 4. S2 and S3 5. S1 and S4 6. *Limit to Human* |
| GWAS Catalog | Terms Searched:   - Cannabis - Cannabis dependence - Marihuana - Marijuana - Cannabinoids - Hash - Kush - Weed - Pot - THC - CBD |
| GWAS Central | Terms Searched:   - Cannabis - Cannabis dependence - Marijuana - Marihuana - Cannabinoids - Hash - Kush - Weed - Pot - THC - CBD |
| NIH Database of Genotypes and Phenotypes | Terms Searched:   - Cannabis - Cannabis dependence - Marijuana - THC - Marihuana - Cannabinoids - Hash - Kush - Weed - Pot - CBD |
